# Attention and counter-framing in the Black Lives Matter movement on Twitter

**DOI:** 10.1057/s41599-022-01384-1

**Published:** 2022-10-12

**Authors:** Colin Klein, Ritsaart Reimann, Ignacio Ojea Quintana, Marc Cheong, Marinus Ferreira, Mark Alfano

**Affiliations:** 1 The Australian National University, Canberra, ACT Australia; 2 Macquarie University, Sydney, NSW Australia; 3 University of Melbourne, Melbourne, VIC Australia

**Keywords:** Complex networks, Cultural and media studies, Philosophy

## Abstract

The social media platform Twitter platform has played a crucial role in the Black Lives Matter (BLM) movement. The immediate, flexible nature of tweets plays a crucial role both in spreading information about the movement’s aims and in organizing individual protests. Twitter has also played an important role in the right-wing reaction to BLM, providing a means to reframe and recontextualize activists’ claims in a more sinister light. The ability to bring about social change depends on the balance of these two forces, and in particular which side can capture and maintain sustained attention. The present study examines 2 years worth of tweets about BLM (about 118 million in total). Timeseries analysis reveals that activists are better at mobilizing rapid attention, whereas right-wing accounts show a pattern of moderate but more sustained activity driven by reaction to political opponents. Topic modeling reveals differences in how different political groups talk about BLM. Most notably, the murder of George Floyd appears to have solidified a right-wing counter-framing of protests as arising from dangerous “terrorist” actors. The study thus sheds light on the complex network and rhetorical effects that drive the struggle for online attention to the BLM movement.

## Introduction

The online platform Twitter has played a central role in the Black Lives Matter (BLM) movement (Tufekci, 2017). Twitter has been used both to draw attention to the movement’s core ideas and to mobilize supporters for protests (Ince et al., 2017). In a recent review, Dunivin et al. (2022) have suggested that increased online attention has made the movement widely visible. Although the #BlackLivesMatter hashtag was first coined in 2013 as a response to the acquittal of George Zimmerman in the shooting death of Trayvon Martin (Lebron, 2017), the murder of George Floyd by police in May 2020 catalyzed the movement both online and on the streets. Twitter played a vital role in that increased engagement.

Wu (2017) notes that “…in the battle for our attention, there is a particular importance in who gets there first or most often.” Yet speed and endurance can dissociate. Rapidly mobilized attention can rapidly fade, while a suitable framing can prolong moderate engagement. Tufekci (2017) argues that left-wing activism has effectively used Twitter to gain rapid attention, but struggles to maintain that message across time. More generally, Twitter is part of the “economy of attention” (Hendricks and Vestergaard, 2019; Simon, 1971). Particular tweets and general issues compete for scarce attentional resources.

Downs (1972) argued that some topics go through a characteristic “Issue-Attention Cycle.” Fervent attention is kicked off by a high-profile event, which opens a window for change before being followed by pessimistic counter-reactions and a fade from public view. The ability to bring about lasting change depends on how much can be done during the period of heightened awareness—which in turn depends on how long that period can be maintained. Downs identified three conditions that make an issue particularly likely to go through this cycle (Downs, 1972, p41ff): it involves a minority population, solving the issue requires dealing with structural considerations that advantage a majority, and the peaks of attention tend to be tied to shocking but transient events. All three conditions are met by BLM. Hence attentional dynamics are key to understanding BLM’s impact: change is more likely the longer it can dominate the awareness portion of the issue-attention cycle.

Yet attention alone is not sufficient for change, as awareness of a movement is not necessarily agreement with its aims. Twitter contains a substantial right-wing presence (Freelon et al., 2020), many of whom are unsympathetic or downright hostile toward the grievances expressed by BLM activists (Keib et al., 2018; Ray et al., 2017). The ability of Twitter to recontextualize and repurpose information makes it a powerful tool for framing BLM—that is, to influence "the context within which an issue, opinion, or claim is made” which in turn "influences our understanding of and attitudes towards it” (Benkler et al., 2018, p. 10). Previous work suggests that even though frames enclose and affect virtually all aspects of our political lives (Scheufele, 2000; Shah et al., 2001), their implications are especially pronounced in the context of social movements (Snow et al., 1988, 1986). One reason for this is that the success of such movements is largely contingent on public perception, which is in turn sensitive to how a movement presents itself and is presented by its opponents (Edwards and Arnon, 2021). By opting for a particular frame, a movement articulates its identity, grievances, strategies, and ambitions in ways that are more or less amenable to public opinion (Ince et al., 2017; Winstead, 2017). As public opinion is fickle, even the most carefully chosen frames are contestable (Riker, 1986).

The present study examines attentional and framing dynamics on Twitter in the context of the Black Lives Matter movement. Insofar as competing frames vie for both attention and approval, we suspect that online social movements will not only be responsive to attention-grabbing events such as police shootings and protests, but also attempt to incorporate those events within an enduring and compelling narrative. In the context of BLM, and the killing of George Floyd in particular, this leads to two research questions (RQ1 and RQ2):

1. How sensitive is the online attention of different groups to protests (and vice versa), and how quickly does that attention decay?
2. How do different groups talk about BLM, and how did that change in response to the murder of George Floyd?

We hypothesized, based in part on the work of Gunn et al. (2018) on responsiveness of politically relevant searches after mass shootings, that RQ1 would reveal a pattern whereby politically left-leaning accounts would show a rapid but more transient response to protests, while politically right-leaning accounts would show a smaller but more sustained response partly driven by reaction to the left. While Gunn et al. (2018) don’t explicitly touch on political affiliation, previous work finds that both gun-ownership and attitudes toward gun-control are strongly sorted along partisan lines, with Republicans generally opposing stricter legislation (Wozniak, 2017) and owning more firearms (Parker et al., 2017). Inasmuch as attitudes toward BLM are polarized along similar ideological cleavages (Alfano et al., 2022; Drakulich and Denver, 2022), and given that both mass- and police-shootings are violent, shocking events, it stands to reason that patterns in responsiveness to police killings will coincide with partisan identification.

For RQ2, we hypothesized that there would be differential framing of BLM narratives, particularly post-Floyd when a pro-police narrative would be more difficult to sustain, but our goals were exploratory with respect to the content of those framing narratives and how they changed.

We approached these questions by using using a large corpus of tweets about Black Lives Matter collected over the whole of 2020 and 2021. We use a mixed-methods, data-driven design to examine different groups in online discourse and the topics with which they engage. Our work builds on a number of recent analyses that employ similar methods, including a comprehensive set of topic models published by Giorgi et al. (2022). In so doing, the current paper contributes to the growing body of work on Black Lives Matter (Crenshaw et al., 2015; Gallagher et al., 2018; Lebron, 2017), the broader literature on online social movements (Hara and Huang, 2011; Harlow, 2012; Tufekci, 2017), and the still nascent study of attentional dynamics in virtual environments (Freelon et al., 2020; Gunn et al., 2018; Hendricks and Vestergaard, 2019).

## Methods

### Data collection

Based on an initial snowball sampling conducted in 2015 of words, phrases, and hashtags associated with the Black Lives Matter movement, we queried the Twitter Streaming API with a series of Black Lives Matter-related keywords, hashtags, and short expressions (see SI Section 1 for more detail, as well as information about code and data availability).

Tweets can fall into one of three categories: original tweets (which can either be de novo or comments on other tweets); quote tweets, which reference and comment on a tweet; and retweets of either original or quote tweets. We examined original tweets and retweets that occurred within 2020 and 2021. Note that a retweet can occur an arbitrarily long time after its parent tweet, though in practice the vast majority of retweets are to very recent tweets. We omitted quote tweets: it is often difficult to tell whether they represent endorsement or criticism, and the text itself can be difficult to interpret without the context of the original tweet.

Over 2 years we collected ~118.7 million tweets of all types. Our access to the Twitter API was disrupted from July 24th through August 8th 2020. We include tweets from that time period if they were later retweeted. These missing days are included in qualitative analyses below, but omitted from quantitative ones. Additionally, we are missing data on pure retweets from May 31, 2020, which we suspect (but cannot confirm) was part of an effort by Twitter to mitigate certain kinds of network effects in the wake of Floyd’s murder. As retweets were only used to build the group network but not as part of the subsequent analysis, the missing retweets should have little effect.

We made no attempt to filter real users from bots. It matters little for our hypotheses whether online dynamics are driven by humans alone or by a combination of humans and bots, so long as a sufficient amount of content is generated by humans. Both humans and bots amplify attention, and so both are appropriate analytic targets.

To examine the real-world causes and consequence of BLM, we used data on BLM protests. Protests are useful both as a proxy for real-world events (such as instances of police brutality) and as a consequence of online social organizing. We examined data on the number of protests per day in the US. Protest data was sourced from the Armed Conflict Location & Event Data Project (ACLED) (Raleigh et al., 2010; see also SI Section 1). Note that the ACLED database only reports the number of protests that occurred during the period in question, not the size. While it would be informative to know exactly how large these protests were, data about protest attendance is both hard to come by and notoriously unreliable. That said, polls conducted by several reputable institutions during June 2020 indicate that anywhere between 15,000,000 (Parker et al., 2020) and 28,000,000 (Hamel et al., 2020) people attended BLM protests in the span of just a few weeks. More important with respect to the current study is that, within certain bounds, the frequency at which protests take place may be more informative than the exact number of people in attendance. For frequency tells us something about diachronic engagement, which is, at any rate, unlikely to be sustained without relatively high and consistent levels of turnout and support.

### Network creation and clustering

To examine social connections, we first generated a *retweet network* (Alfano et al., 2022; Sullivan et al., 2020): a weighted undirected network in which nodes represent authors and the weight of an edge represents the number of times that one user retweeted the other. Bare retweets (rather than quote tweets) almost always indicate endorsement of content (Metaxas et al., 2014), so looking at patterns of retweeting is a good way to reveal affiliative networks. Self-retweeting was discarded. The original retweet network consisted of ~18 million authors with ~98 million directed edges representing ~118 million total retweets. The vast majority of these authors were connected to each other by a single retweet. We further culled the network by eliminating edges with a weight ≤3 and then taking the largest connected component ( ~655 k nodes, ~1.7 M edges) for further analysis.

To find users who clustered together in their retweet behavior we used the Leiden community detection algorithm (Traag et al., 2019) as implemented in *igraph* (Csardi and Nepusz, 2005) and the Python *leidenalg* package (Traag, 2020; Traag et al., 2019). We kept clusters containing at least 5% of the original nodes. The remainder of the analyses were conducted on authors who fell into one of these clusters.

### Autoregression

To examine the first research question, we conducted a timeseries analysis of the number of tweets per day by each group. An autoregression (AR) predicts the value of a variable on a day as a function of time-lagged values of both that variable and other variables. Autoregression was used successfully by Gunn et al. (2018) to examine online timecourse data about shootings and gun control as a proxy for political engagement. We looked at two autoregressions:

- **Model I:** “**Naive**” **AR** The first autoregression model considered only the number of tweets per day for a group, and predicted these as a function of their own lagged tweets plus lagged protests per day as an exogenous variable. This estimated how responsive groups were to external events (using protests as a proxy) and how quickly that responsiveness decayed.
- **Model II:** “**Full**” **Vector Autoregression (VAR)** The second analysis used vector autoregression (VAR) and considered the responsiveness of *both* group tweets and of protests as a function of lagged versions of each. The full VAR model differs from Naive AR in two respects: it treats protests as an endogenous variable (and so protests might be influenced by tweets as well as influencing them), and allows for the possibility that groups might influence one another.

The number of tweets per day varies by orders of magnitude over the course of the study, and the number of protests per day is on a smaller scale (and occasionally 0). To normalize the data to a standard scale we used the *z*-score of tweets and protests per day (calculated separately for each). When interpreting the results, therefore, the coefficients should be read as showing the effect of a one standard deviation (SD) variation in the corresponding variable in terms of SD units. The transformed scores were stationary using the AD Fuller test (see SI Section 3 for details). Both simple autoregression and VAR were calculated using the Python *statsmodels* package (Seabold and Perktold, 2010). Transformed protests were treated as an exogenous variable for the first analysis, and as an endogenous variable for the second. Data for the missing period was dropped from the analysis.

### Topic modeling

To address the second research question, we turned to topic modeling. Topic modeling provides a useful way to give a high-level summary both of the themes in a corpus and of the particular content of individual documents. Allen and Murdock (2022) suggest that topics can be fruitfully thought of as indicating the contexts in which individual authors present and understand their own work. In topic modeling, documents $d\in {{{\mathcal{D}}}}$ are represented as distributions over topics and topics $t\in {{{\mathcal{T}}}}$ are distributions over words $w\in {{{\mathcal{W}}}}$. In Latent Dirchlet Analysis (LDA), the weights are obtained by Bayesian updating, taking as priors Dirichlet distributions (the conjugate prior of the multinomial distribution). The matrix of frequencies of words per document with dimension *D* × *W* is decomposed into a matrix of dimension *D* × *T* representing the distributions of topics per document, and a matrix of dimension *T* × *W* representing the distributions of words per topic. As other unsupervised methods, topics allow for a lower dimensional summary of the data.

We examined original tweets made in 2020 and 2021 by each author who had been assigned to one of the main clusters. Note that while the clusters were made on the basis of retweet behavior, the tweets used need not have been retweeted to be included. Tweets were preprocessed and aggregated by author before fitting the model (see SI Section 1 for details about preprocessing). Each document in our corpus consisted of a single author’s aggregated preprocessed tweets. Aggregating author tweets for the initial topic model gives a better sense of the interests of individual authors (Alfano et al., 2022), as well as avoiding problems caused by generating topic models on very short fragments of text.

Latent Dirchlet Analysis was performed using the default parameters in *scikit-learn* 1.02. Our research question preferred a relatively small number of topics. To choose the specific topic number *k* we first built models for *k* = {3, 6, 9. . . 45}. For each model, we then tested the ability of a linear discriminant analysis to classify authors into clusters, taking the mean performance over 10 75/25 random train-test splits. Each model performed well above chance, with a clear improvement at *k* = 24 and diminishing returns thereafter (see SI Section 4). We thus used *k* = 24 for further analysis.

To give a qualitative picture of each group’s concerns and how they changed over time, the fitted topic model was then used to transform each individual tweet from each author. Each tweet was assigned to the topic corresponding to its maximum loading, and the proportion of tweets per topic per day was calculated for each group.

## Results

### Descriptive results

The clustering of the retweet network produced three clusters that were at least 5% of the original network, jointly covering 50% of authors in the the graph. To characterize the groups, we manually examined the top posters in each group, ordered by PageRank. Table 1 shows some of the top posters for each group, organized by our proposed descriptive label.

**Table 1:** Representative sample of top posters for each group, post-clustering, and imputed name.

Group	%/(*n*)	Top users
Right	23.3 (152,367)	MrAndyNgo RealCandaceO stillgray dbongino RealJamesWoods charliekirk11 marklevinshow w_terrence gatewaypundit MarkDice DineshDSouza realDonaldTrump johncardillo PrisonPlanet RyanAFournier larryelder BernardKerik BreitbartNews JackPosobiec ElijahSchaffer mitchellvii BrandonStraka ChuckCallesto CassandraRules DiamondandSilk prageru theangiestanton TheRightMelissa MattWalshBlog no_silenced DailyCaller WayneDupreeShow DC_Draino theblaze JesseKellyDC mtgreenee RudyGiuliani KarluskaP
Center-Left	20.6 (134,795)	AttorneyCrump _SJPeace_ TomthunkitsMind common BerniceKing ACLU CNN kylegriffin1 TalbertSwan ajplus mmpadellan nowthisnews JoyAnnReid JuddLegum thehill davidmweissman TIME QasimRashid RBReich SpeakerPelosi KamalaHarris shannonrwatts ananavarro ABC Blklivesmatter RawStory mhdksafa NBCNews CBSNews MSNBC Independent nytimes RexChapman
Activist	6.2 (40,846)	YourAnonCentral YourAnonNews KenidraRWoods_ 4theculture____ LatestAnonNews elijahdaniel YourAnonRiots PalayeRoyale vestergaah SebastianDanzig snowlions NoNameoN_A YourAnonCentril Subtronics AnonOpsSE YourAnonS0u1 Michael5SOS NrSomething NiaLovelis AnonOpUSA ASB_Breaking notices2020 5sosworldalerts Kellinquinn TDoRinfo echoeslrh

% indicates percentage of total number of nodes (assigned or not). To protect the privacy of users, we only list institutional/organizational accounts, verified accounts, and accounts that have been suspended or deleted.

The groupings we found were in line with previous work, reflecting a general division between pro- and anti-BLM communities (Araque et al., 2020; Gallagher et al., 2018; Ince et al., 2017; Ray et al., 2017), as well as more fine-grained distinctions within these two camps using different timeslices and clustering methods (Alfano et al., 2022; Stewart et al., 2017). Note, for example, that the Right group contains the account of then-president Donald Trump and right-wing standards such as Breitbart news. Similarly, the Center-Left group contains mainstream Center-Left news outlets, the ACLU, and prominent civil rights lawyers. The Activist group is more mixed, but includes numerous accounts that self-describe as activist as well as a number of online left-wing news outlets.

**Fig. 1:**
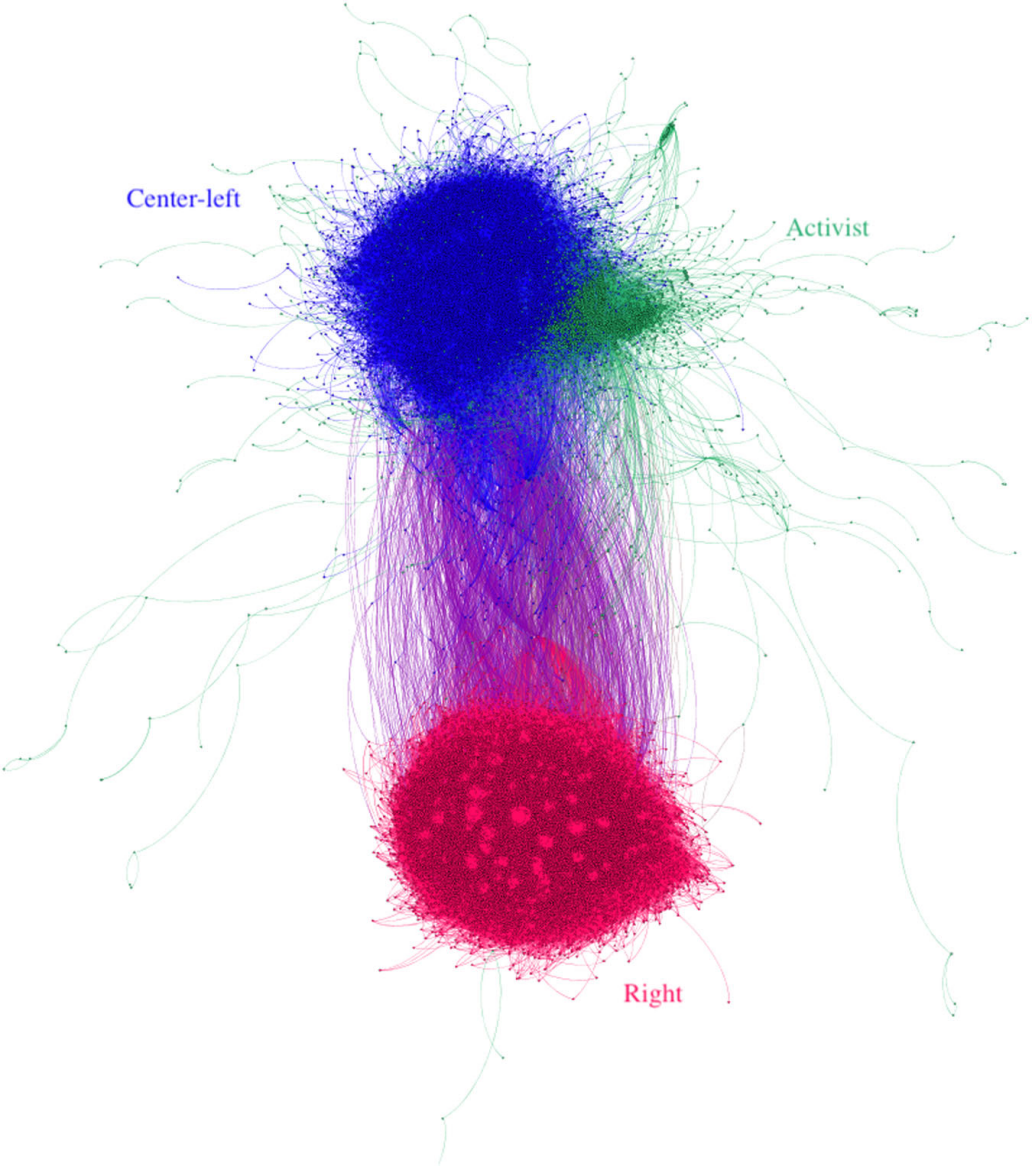
Retweet graph with groups labeled. Nodes represent authors, edges weighted by number of retweets of one author by the other. Only the top 25% of retweeting nodes in each cluster are shown; apparently solitary pockets are connected to the others in the group on the strength of weak ties here omitted. Layout by Gephi’s *forceatlas2* algorithm.

Figure 1 visualizes the subgraph of the retweet network containing the top authors in the three largest clusters. It shows a familiar picture of political polarization: by and large the Right retweets the Right and the Left the Left, with a few bridge accounts connecting both. The Activist group is, unsurprisingly, closely aligned with the Center-Left, but forms a distinct cluster including smaller pockets with unusual alignments.

**Fig. 2:**
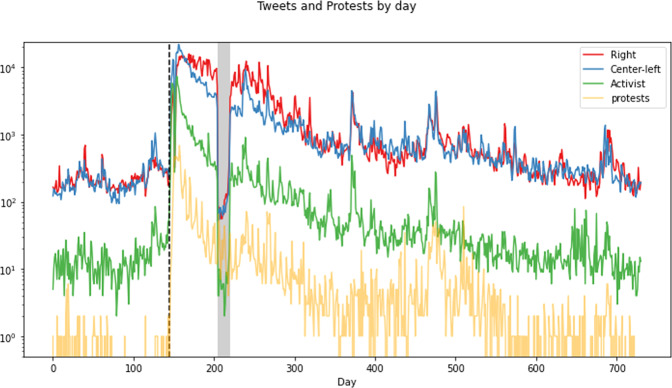
Original tweets and protests. *y*-axis is on a log scale. Black dashed line shows the date of George Floyd's murder. Gray bar shows period of disrupted data collection.

As Fig. 2 shows, both Center-Left and Right authors were engaged in BLM discourse from the beginning of data collection, with a substantial number of tweeters on both sides of the political divide. As one would expect, there was a significant spike in activity after the murder of George Floyd, with daily activity jumping roughly two orders of magnitude (see SI Section 2 for similar figures on rewteets and unique authors per day). Previous work shows that the killing of Michael Brown on August 9th 2014 generated a similar spike, albeit at a lower order of magnitude (Giorgi et al., 2022; Ince et al., 2017).

### Autoregression

The naive autoregression analysis showed differences between groups both in responsiveness to protests and in the rate at which tweet activity would be expected to decay over time after an initial shock (either a 1SD increase in tweets or a 1SD increase in protests). Letting *s* be the decay coefficient and *r* the protest coefficient, Activists were most responsive to protests but with a faster decay (*s* = 0.76; *r* = 0.14), while the Right were less reactive to tweets but had a much slower decay (*s* = 0.92; *r* = 0.06). Center-Left accounts were somewhere in the middle (*s* = 0.88; *r* = 0.10).

**Fig. 3:**
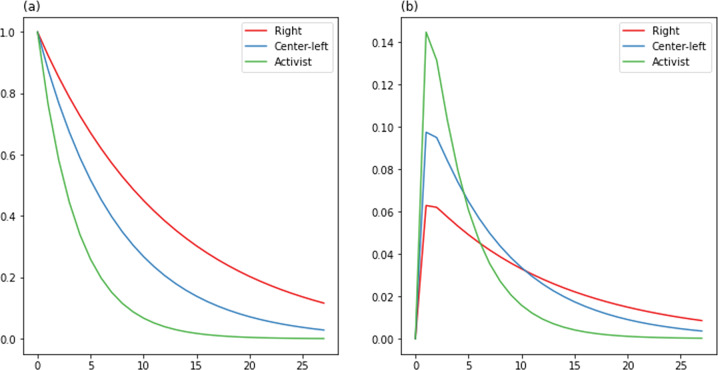
Impulse response for naive autoregression. Projected impulse response over 28 days after a 1 SD shock to **a** within-group tweets and **b** protests. The *x*-axis shows day, *y*-axis predicted SD change in total tweets.

To visualize our results, we plot the impulse response functions (IRFs) for both Models I and II. IRFs analyze “interactions between variables in a vector autoregressive model...” by representing “reactions of the variables to shocks hitting the system” (Lütkepohl, 2016). As the left half of Fig. 3 shows, the effect of a 1 SD-deviation in tweets lasts substantially longer for Right posters than others. Conversely, the right half of Fig. 3 shows that a 1SD deviation in protests predicts a much higher peak from Activists, but this effect decays more quickly, with the effect on the Center-Left larger by six days after, and that on the Right by day 12. Practically speaking, this means that Activists tend to show much more activity in response to protests, but that this activity dies off relatively quickly; the Right, by contrast, doesn’t respond as strongly to protests but their activity is sustained for a much longer time.

**Fig. 4:**
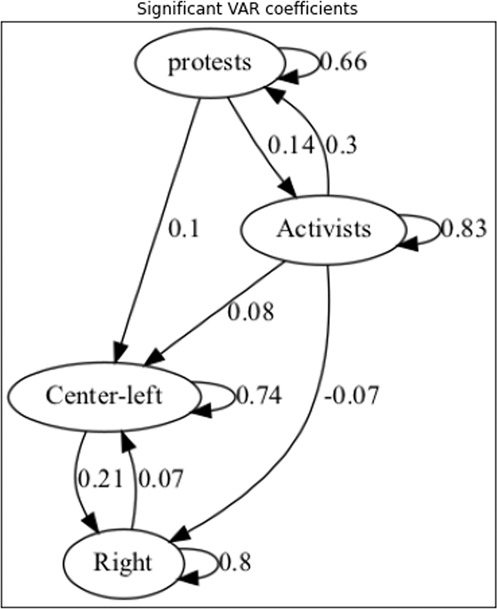
Coefficients for full VAR model. Lag-1 coefficients for full VAR model significant at alpha = 0.01 (uncorrected).

The full VAR model (Model II) paints a more nuanced picture. Figure 4 depicts the statistically significant coefficients for each variable (the full table can be found Section 3 of the supplemental material while Supplemental Fig. S5 shows the impulse response functions for each pair of variables).

Several results are worth noting. First, the decay coefficient for Activists is now highest of all. Indeed, as the full IRF figure shows, a 1-SD increase in tweets for Activists would have a statistically significant effect as far out as day 20, while the same change in other groups would drop below significance by day 10. The sensitivity results to protests remain the same (though the coefficient for the Right is not statistically significant).

Model II shows more complex interactions between groups and protests. Notably, the Activists appear to have a relatively strong effect on protests (whereas neither Right nor the Center-Left tweets make an appreciable difference). This fits with previous work showing that activity on Twitter can be predictive of later protests (Bahrami et al., 2018; Cadena et al., 2015), and that the predictive value of Twitter for protests often depends on the specific situation of authors (Korolov et al., 2016; Mooijman et al., 2018). The effect we see might be due to direct influence (calling for protests, calling attention to injustices, and so on), or to an indirect sensitization effect.

On the other hand, tweets by the Center-Left have a reasonably strong influence on the Right, while there is a weaker influence on Center-Left tweets from both Activists and the Right. This means that Activists drive activity both in the Center-Left and, after a delay due to their direct dampening effect, the Right. There is no converse effect of note on Activists, however.

The net effect of this influence, as seen in left column of figure of the full IRF figure, is to reproduce the pattern seen in the naive autoregression. A 1-SD change in protests causes a rapid peak in the Activists, and a smaller, slower-to-peak and slower-to-decay response in the Right. The latter response is predicted not just by the initial protests, but also by accumulated influence from the Center-Left.

In sum, the strongest predictor for each group, on either model, is its own activity on the previous day. There is relatively limited interaction between Activists and the Center-Left, and a slightly negative influence between Activists and the Right. System shocks appear to have a rapid and strong influence on left-wing political positions, followed by a slower but more sustained right-wing response that appears to be partly driven by a reaction to the initial left-wing response.

### Topic modeling

The full 24-topic model is presented as Table 2. There are a number of topics that express distinctly pro- and anti-BLM viewpoints, as well as neutral topics about protests and more general “discursive” topics. Some topics (such as #3) represent features of BLM discourse that have been present from early on, while others (such as the George Floyd and Breonna Taylor focused #1 and the QAnon-inflected #18) reflect developments arising in 2020 and beyond.

**Table 2:** Top words for each LDA topic. Model fitted on aggregate author tweets.

#	Top words
0	blacklivesmatter georgefloyd breonnataylor black icantbreathe
1	matter black life live say white people movement racist support
2	blm antifa terrorist marxist riot black organization support
3	knee flag stand anthem nfl player national kneel watch sport
4	cop kill police murder officer shoot arrest man year black old
5	covid mask protest wear stay coronavirus news pandemic home
6	democrat biden party money fund joe soros democratic obama donation
7	vote trump american america president republican voter country
8	trump protest protester capitol say white sign police gun peaceful
9	medium social month america tell justice family remember really
10	police brutality protest black racism stop protester america
11	blm donate share retweet need facebook help joebiden face hate
12	history right human blacktwitter racism time change fix rap
13	blue help state red bring send business georgia donate flip
14	policebrutality video follow new watch anonymous check break
15	justice sayhername black today sign day george floyd fight breonna
16	people just say think white know make right fuck want racist
17	black racism make people issue movement end community white
18	trump maga usa kag wwgwga qanon patriot walkaway supporter lawandorder
19	police protest black protester trump street city activist mob
20	bluelivesmatter thinblueline say push slap jail report catholic
21	veteran update latino asian south mexican indian crash new american
22	alllivesmatter whitelivesmatter race color racist die skin alllivesmattter
23	backtheblue police god sayhisname officer law thank enforcement

The topics identified in our model are broadly consistent with both general theories of social movement frames (Benford and Snow, 2000; Snow et al., 1986) and several recent studies that focus specifically on Black Lives Matter (Gallagher et al., 2018; Giorgi et al., 2022; Ince et al., 2017; Ray et al., 2017). In line with general theory, we find that topics cluster into three broad categories: serving diagnostic, prognostic, and motivational functions respectively. For instance, in emphasizing police brutality and racial injustice, several topics (e.g., 2, 14, 15) take on a distinctly diagnostic role: articulating grievances and stressing the need for change. Other topics (e.g., 6, 10, 11) transform these grievances into action plans: calling for protests and other forms of political resistance. Previous work suggests that these prognostic topics played an important role in organizing BLM activism (Keib et al., 2018). Finally, to compel the community into action, BLM, alike other movements, enlists various motivational frames (e.g., 0, 1, 12): mobilizing activists by naming victims, naming perpetrators, and foregrounding the group’s collective history and identity (Brown et al., 2017; Winstead, 2017).

Several of the themes that we identify are also comparable to those found in previous studies, including studies that focus specifically on counter-frames. Topics 20, 22, and 23 for instance corroborate the results of Ince et al. (2017), Gallagher et al. (2018), and Giorgi et al. (2022), all of whom find that #AllLivesMatter and #BlueLiveMatter are dominant frames for articulating anti-BLM attitudes. Worth noting is that our method for identifying topics differs somewhat from previous work. For instance, in contrast to Giorgi et al. (2022), we include *both* retweets and original tweets rather than original tweets only. Relatedly, our topic models reflect themes that are dominant within particular *retweet networks*, as opposed to themes that emerged around specific hashtags. A notable upshot of our approach is that it sheds lights on several themes missed by other models—e.g., topic 2, which portrays BLM activists as “terrorists”. To the best of our knowledge, Ray et al. (2017) are the only other authors to pick up on this topic, ablbeit in the context of the murder of Micheal Brown. We will return to how and why this frame (re)emerged in the context of George Floyd’s murder.

**Fig. 5:**
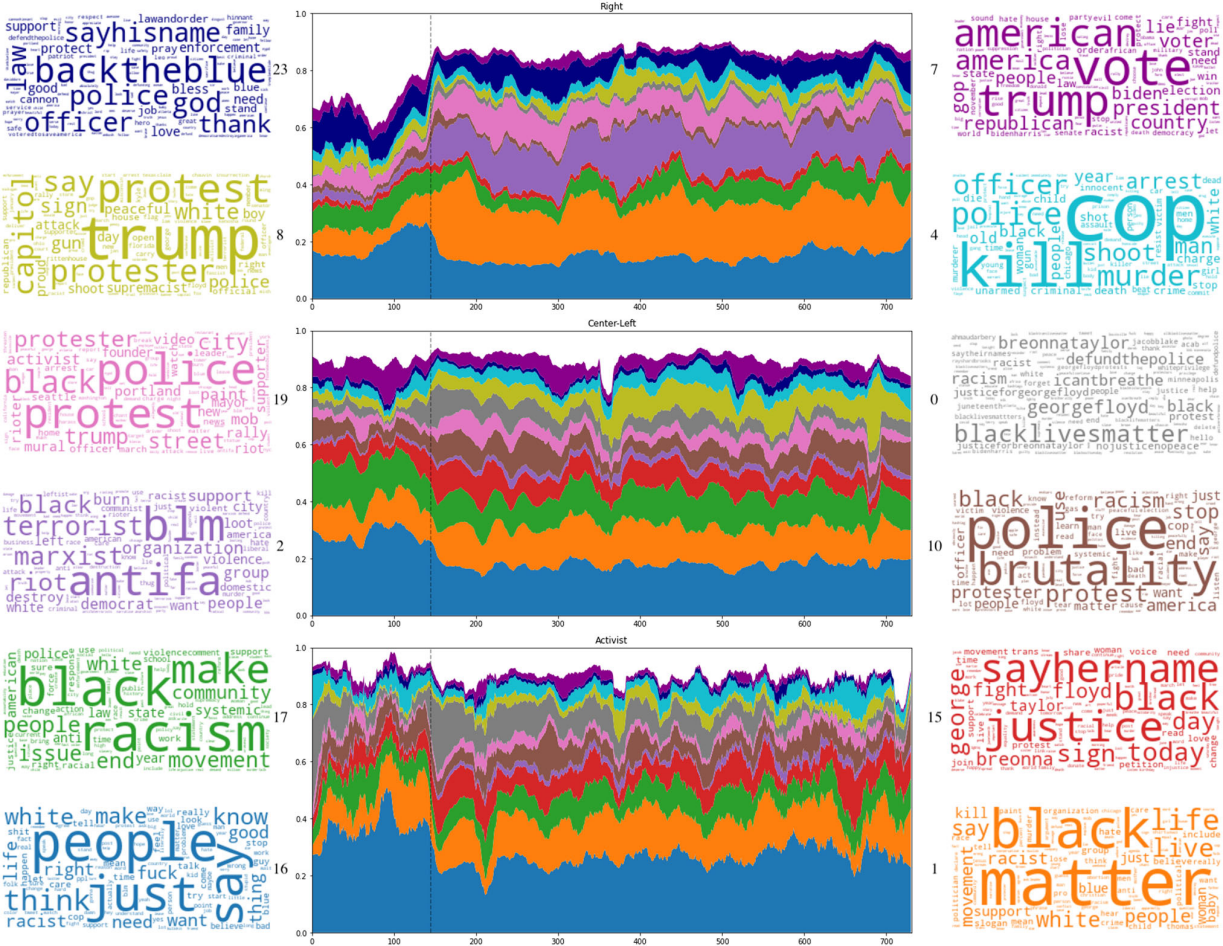
Proportion of tweets in each cluster with a maximum loading on each of the top 12 topics. Colors correspond to wordclouds representing each topic; topic number is next to each word cloud. Vertical line is date of George Floyd’s murder. Timeseries for each topic smoothed using a 15-day linear Savitzky-Golay filter (Savitzky and Golay, 1964).

Figure 5 visualizes the top 12 most common topics by tweet for each cluster, along with wordclouds representing the corresponding topics. The dashed vertical line indicates the murder of George Floyd, which made a notable difference in both quantitative and qualitative aspects of online BLM discourse.

We note several patterns. First, both Center-Left and Activist posters had relatively consistent levels of engagement across topics. The notable difference, coinciding with the murder of George Floyd, was an increase among Activists in the proportion of tweets that loaded on topic #0 (gray), which contains a number of Floyd-related terms. This was at the expense of more generic topics—notably the discursive topic #16 (blue) and the general topic #1 (orange), suggesting a more focused discourse.

There are further differences between the groups. The Center-Left appeals more consistently to topic #17 (green), which appears to focus on systemic racism. The activist loadings are more variable over time even with smoothing, consistent with a greater responsiveness to external events. (Note the sudden dip just shy of 400 days that coincides with the Jan 6 2021 Capitol Building insurrection; there various minor, mostly Trump-related, topics briefly take over.) The other notable difference between the Center-Left and other groups is a greater willingness to discuss both protests in the context of Trump (topic #8, gold) and the election more generally (topic #7, dark purple) than either Activist or Right accounts, suggesting a belief that it might be a winning election issue.

The Right tweets differ from the others in several notable ways. On the one hand, the murder of George Floyd increased engagement with BLM issues generally (c.f. also Fig. 2) This is reflected in the topic modeling, where the proportion of tweets devoted to topic #1 (orange) increases dramatically. Before Floyd there was a larger concentration of BLM-themed tweets on other tangential topics—especially the immigration- and military- focused topic #21 and the COVID-themed topic #5. These become almost entirely absent afterwards. (Not pictured in the graph as they are not part of the top 12 topics, but see SI Section 4 for details). This is consistent with the results of Dunivin et al. (2022), and indeed suggests that the BLM movement has garnered increased attention across the political spectrum. The shift away from apparent distractor topics (such as immigration and COVID) and towards general discussion among the Right suggests more consistent and coherent engagement.

On the other hand, the *manner* in which the Right talks about BLM suggests a more complex narrative. Most notable is the rapid increase in topic #2 (purple), which contains words connecting the BLM movement to Marxism, antifa, terrorism, and other bugbears of US right-wing discourse. This topic is barely present in other groups. Moreover, it plays only a minor role in the Right before Floyd but becomes one of the standard framings after. Pro-police rhetoric (topic #23, dark blue) is slightly diminished immediately following Floyd’s murder, reappears in roughly the same proportions shortly thereafter, but loses ground relative to topic # 2.

Hence, after Floyd’s murder, we see what appears to be a permanent shift in the dominant framing for Right-wing tweets. Before Floyd, pro-police rhetoric plays an important role; after, the pro-police tweets are swamped by the “antifa terrorist” frame.

**Fig. 6:**
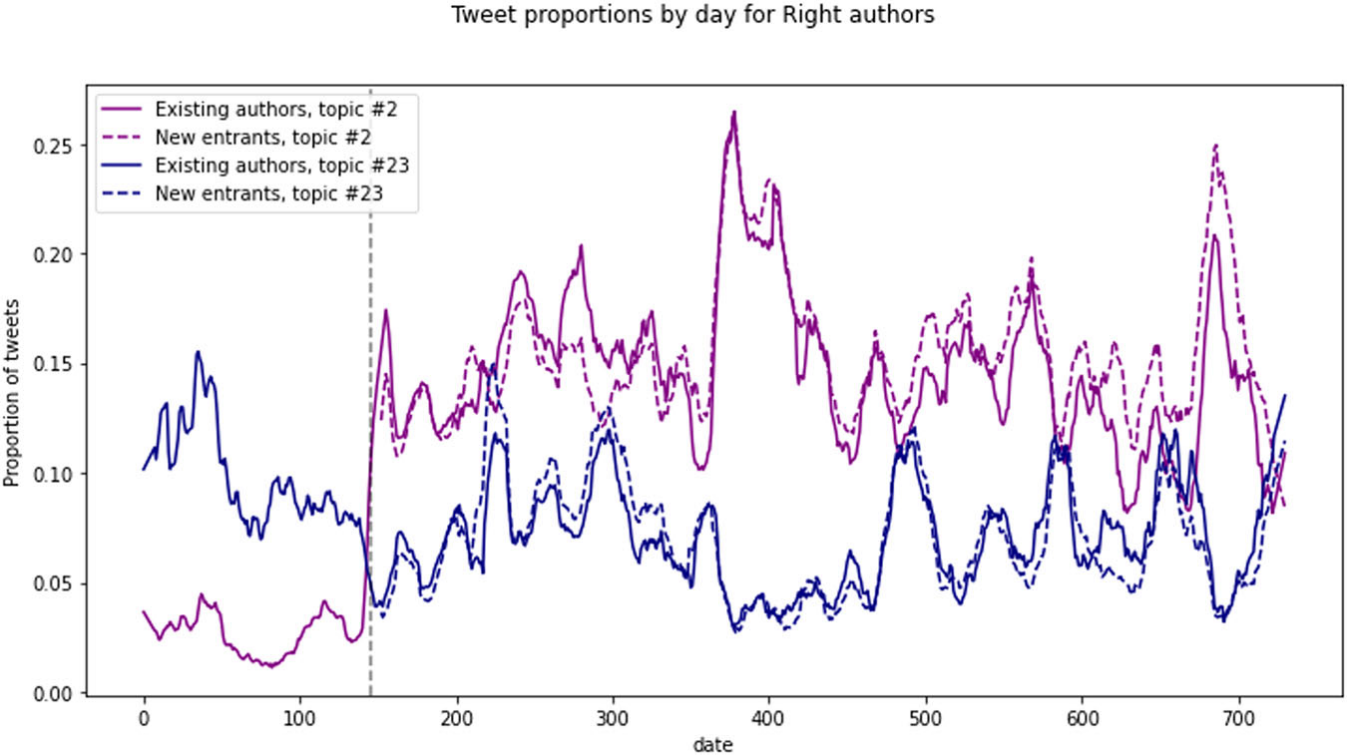
Proportion of Right tweets with maximum loading on two selected topics, distinguishing authors who posted at least once before Floyd’s murder and newcomers. Topic #2 is the “Antifa terrorist” framing, #23 is a pro-police topic. Timeseries for each topic smoothed using a 15-day linear Savitzky-Golay filter.

The frame shift for the Right authors is so pronounced that we performed a further ad-hoc analysis to examine its causes. To rule out the possibility that this shift was driven by an influx of new participants with pre-existing “anti-antifa” sentiments, we split the Right into authors who made at least one tweet before Floyd’s murder (*n* = 27,176) and those who didn’t appear in our dataset until after that date (*n* = 82,043) and re-ran the same proportional analysis as depicted in Fig. 5. The results in Fig. 6 suggest that the two groups of Right authors are basically indistinguishable. The “antifa terrorist” framing of protesters was equally popular with both pre-existing authors and newcomers alike, suggesting a global shift in framing by the Right rather than discursive infiltration by a specifically “anti-antifa” contingent.

A final point is worth noting. There are three topics that focus on protests, but which appear to have different valence. The Right favors topic #19 (pink), which is concerned with protests but appears to be focused more on the police responses to protestors and on specific cities. Activists favor topic #10 (brown), which has a distinct focus on police brutality. The Center-Left appears to incorporate both, as well a topic #8 (gold), which incorporates a heavy focus on Trump as well. This suggests that our model is sensitive not just to discussion of protests, but to differences between the ways groups frame protests.

## Discussion

### General remarks

Our work suggests that Activists and right-wing tweeters represent two different poles of a speed/endurance tradeoff. The former favors focused, agile, and responsive attention to immediate events; the latter favors slow and sustained attention to a consistent alternative framing.

These results are consistent with previous work showing differential dynamics of online attention among different political groups. Online searches about gun control for instance spike after mass shootings in the US, which drives a reactive, slower, but longer-lasting interest in searches about gun purchasing among right-leaning individuals (Gunn et al., 2018). By the same token, Lee et al. (2022) recently showed that left-leaning politically controversial videos on YouTube (including videos about BLM) tended to get more views, but that right-leaning videos overall garnered more tweets and attention over a longer period of time.

Why do the two political poles differ? Multiple mechanisms may be at work; our results shed light on several of them. First, our VAR analysis showed an asymmetric influence between Center-Left and Right tweets. This suggests that the asymmetry in attention is partly driven by the Right reacting to political opponents more than the reverse. This is consistent with the recent finding of Wu and Resnick (2021), who found that conservatives are much more likely to engage with left-leaning videos on YouTube than the reverse. Indeed, one conclusion suggested by both the VAR and the topic models is that the Right doesn’t seem to respond directly to protests. Instead, they respond indirectly to protesters (Activists) and directly to their (Center-Left) advocates, both of whom are portrayed as threatening American values and institutions. Given that perceived threat is among the strongest predictors of social support for continued repression of aggrieved groups (Edwards and Arnon, 2021), this frame clearly reflects the Right’s strategic interests.

Second, differences in offline structural forces may play a role. Tufekci (2017) has argued that online platforms have made protests at once both powerful and fragile: Twitter is good at mobilizing supporters, but less useful for building the organizational structures that support long-lasting change. Conversely, Schradie (2019) has argued that conservatives have had an advantage on digital media because they are ideologically more hierarchical, and therefore better-poised to promulgate consistent messaging. This argument is consonant with Ray et al. (2017), who note that the online counter-narrative to BLM is controlled by a tightly organized group of conservative ideologues, some of whom have close links with the Republican party. Relatedly, Freelon et al. (2020) note that right-wing tweeters are more willing to work with legacy partisan media to achieve their ends. Finally, Benkler et al. (2018) argue that right-leaning mass media tend to be more willing to disseminate outlandish claims and continue to recycle those claims even after they have been debunked, therein keeping attention fixed on certain issues that are consistent with a chosen ideological frame. Benkler et al. (2018) outline a “propaganda pipeline” through which fringe stories on online message boards are picked up by mainstream outlets only to be pumped back into the internet with greater reach and longevity, regardless of veracity. In sum, Right-leaning media may be more willing to beat the drum on particular talking-points, further extending the expected “life-span” of a given news item. Hence these organizational structures might lend inertia to online activity.

A third likely mechanism is the framing effects identified by RQ2. Although the effects of competing frames are familiar from traditional media (Entman, 2007; Scheufele, 1999; Tankard et al., 2001), the decentralized, many-to-many architecture of platforms such as Twitter engenders a kind of “distributed framing” (Ince et al., 2017), where virtually any user can contribute to the dominant narrative. As the theorist Jacques Ellul noted, the work of propaganda is to provide a steady reinforcement or undermining of stereotypes. This process works best if it can latch onto "the fundamental currents of the society it seeks to influence” (Ellul et al., 1973, p. 38). Both framing and counterframing in the Black Lives Matter movement seek to tap into powerful social currents. US society *has* been responsive to matters of racial justice, particularly when injustice is framed as conflicting with other basic beliefs around personal dignity and individual freedom from government interference (Schuman et al., 1997). By contrast, the right-wing counternarrative we find on Twitter taps into long-established fears of the “enemy within” who seeks to undermine a way of life by undermining legitimate authority. The idea that US society is being undermined by a Marxist elite has long played a role in right-wing conspiracy theorizing (Stormer, 1964). More recently, Crimston et al. (2021) found that moral polarization combined with perceived breakdown of the social order, both of which are salient in the context of BLM, increases support for conservative/authoritarian leaders—and, presumably, the hierarchal organizations that Schradie (2019) identifies as key to sustained conservative attention on an issue.

Fourth, attentional dynamics and framing are likely to interact. Social media such as Twitter are especially sensitive to what Ridolfo and DeVoss (2009, 2017) term the “rhetorical velocity” of a piece of content—that is, to the speed at which different groups might promote or repurpose material. The particular affordances of Twitter give content an especially high rhetorical velocity. Retweets allow for rapid spread of information, while hashtags and quote tweets open up the possibility of appropriation (Ince et al., 2017; Ray et al., 2017). Ridolfo and DeVoss (2017) note, for example, how a Trump Hotels promotional tweet became repurposed years later as a vehicle for expressing outrage at Trump’s executive orders. The same dynamic is evident for BLM. For example, previous research has shown the role of the #AllLivesMatter hashtag in initially setting up a counternarrative to BLM, before being partly reclaimed by activists (Gallagher et al., 2018). Indeed, attempts among right-leaning users to draw *attention* away from “Black” and toward “All” or even specifically “Blue” lives are emblematic of the wider framing contest between these two camps, as well as the attentional implications of successful frame appropriation (Atkins, 2019; Carney, 2016; Gallagher et al., 2018).

Fifth and finally, the algorithms underlying Twitter’s promotion and recommendation of tweets is likely itself part of the explanation (Byrd et al., 2017; Cox, 2017). Huszár et al. (2022) provide evidence that the algorithm consistently amplifies right-leaning voices more than left-leaning ones. Algorithmic personalization systems on social media platforms such as Twitter are also tuned to promote engagement (Alfano et al., 2018, 2021; Burr et al., 2018). Hence which tweets get there “first or most often” is driven in part by what gets recommended, which is driven in turn by what others in a network find engaging. A good framing narrative begets recommendations, which begets more attention, which further entrenches a frame.

This frame-entrenchment effect is potentially exacerbated by the fact that algorithms do not promote the same content equally to everybody, but instead classify individual users as differentially more likely to engage with particular kinds of content based on their social ties. Inasmuch as clusters of socially connected users are thus likely to be presented with broadly similar content (Barberá et al., 2015; Cinelli et al., 2021), and that content exhibits particular frames, algorithmic recommendation potentially sets up what, to repurpose a familiar phrase, can be aptly referred to as “frame bubbles”. In much the same way that traditional *filter* bubbles lead to ignorance of alternative viewpoints (Nguyen, 2020; Sunstein, 2018), users trapped inside *frame* bubbles are liable to have their attention algorithmically fixed on only those frames that are consistent with their side of the narrative, leading that narrative to become entrenched further still. Worth adding to this is that algorithmic effects can interact with cognitive phenomena such as the “what you see is all there is” mindset (Kahneman, 2011). As and when this happens, users who are exposed to only one frame—say, the “Antifa terrorist” frame—may come to believe that this is the *only* available or reasonable interpretation of current events. To us, this suggests that the attentional dynamics of BLM discourse on Twitter are embedded within a complex, multi-layered, and potentially iterative framing sequence, where ideological frames are algorithmically filtered in ways that trigger lower-level cognitive framing effects, which in turn feedback into the algorithmic prioritization of particular ideological frames and, in so doing, keep attention fixed on one narrative rather than another.

### Limitations and future directions

Our work is subject to several limitations. The roughly 15-day gap in data collection was unfortunate, though missing data should only be expected to reduce statistical power, and doesn’t appear to have affected the topic modeling in meaningful ways. Our reliance on API sampling rates, however, means that resampling the gap is not viable. Future work might consider verifying our findings using Twitter’s academic API (unavailable when the study started), giving an independent confirmation of these processes.

The groups we examined are relatively large and coarse-grained. They likely consist of multiple subgroups, but our pilot work on retweet networks has found it difficult to reliably identify stable, interesting subgroups. This is likely because broad groups tend to consist of more fluid, shifting coalitions, and so analysis of overall retweet networks either cannot find subgroups or cannot find useful ones. Further work on identifying temporally fluid subgroups would be helpful.

Our use of discrete econometric methods with a one-day grain throws away considerable information, especially in the context of rapid engagement with tweets. We chose this in part to match the timescale of our protest data. Previous work linking tweets to protest behavior has suggested that even hour-level data can be predictive (Mooijman et al., 2018). Future work on engagement might consider more sophisticated continuous models, such as the Hawkes point process approach used by (Lee et al., 2022).

Our study was limited in the sense that it did not collect all tweets by included authors, only those on BLM, and did not look at interactions other than retweeting. Given the asymmetry in online commenting noted by Wu and Resnick (2021), future work might study the extent to which alternative frames emerge in the course of interaction between different groups. Our methods were also not fine-grained enough to identify tweeters who changed their group affiliation over the course of the study. Given the general entrenchment of political attitudes in the US, we suspect that there are relatively few such authors, but it would be useful to have confirmation of this pessimistic conclusion.

Finally, as in most studies, our approach is fundamentally limited in its ability to collect surrounding context or detailed demographic information about tweet authors.

### Conclusion

Our study shows that proponents and opponents of the Black Lives Matter movement show systematically different patterns of attention in the service of different ways of framing the core issues. Yet while it this may appear to be a familiar story of political polarization, we find two less familiar takeaway lessons.

We paid most attention to the two poles of the debate, but there is an important role played by the Center-Left group of tweeters as well. These authors are structurally and ideologically closely allied with Activists, but they have an intermediate attentional dynamic. The media landscape is constantly evolving in response to new technology (Garcia et al., 2019), and it may be that the current partisan divide in technological uptake has yet to reach an equilibrium point. Our work would suggest that traditional media can play an effective stabilizing role, smoothing out rapid fluctuations—and that it can do so regardless of where it sits on the political spectrum.

Second, we note that attentional dynamics might themselves represent an interesting point of intervention on social networks. Most proposed interventions on social networking concern either the content of messages (e.g., filtering misinformation) or the connectivity of the network (e.g., banning users). Both can be difficult to implement and politically controversial. Our work suggests that there is considerable variability in the attentional dynamics of online media as well. As a rough proposal, Twitter could ensure that hashtags that are rapidly gaining attention are presented alongside (less popular) hashtags that offer an alternative interpretation of events. Such a policy might prevent frame entrenchment by ensuring that users become aware of competing frames at an early stage. Alternatively, and perhaps better yet, Twitter could pair clearly partisan or racist hashtags with hashtags that activate superordinate identities, exposure to which has been shown to reduce out-group (Nordlinger, 1972) and cross-partisan animus (Brady et al., 2020; Van Bavel and Pereira, 2018).

In any case, it is important to emphasize that algorithms don’t simply promote tweets: they promote tweets of a certain age, with a certain speed, and with a certain history of visibility. It may be too much to hope for an uncontroversial set of neutral criteria that can moderate attentional dynamics for the overall good, but careful study of the ways in which attentional framing and dynamics interact will be a key to any solution.

## Supplementary information

Supplemental Material

## Data Availability

Python notebooks containing the entire processing chain for the paper along with instructions for adapting other datasets are available at https://osf.io/amv3r/. Also available are a collection of ‘dehydrated’ tweet IDs for both retweets and tweets, suitable for reconstruction using tools such as TWARC.
