# Supplementary material for: Attention and counter-framing in the Black Lives Matter movement on Twitter
Source: Humanit Soc Sci Commun. 2022 Oct 12;9(1):367. doi: 10.1057/s41599-022-01384-1 (PMC9555697; doi:10.1057/s41599-022-01384-1)
Supplement: Supplementary file 1 — Supplemental Material [file 41599_2022_1384_MOESM1_ESM.pdf]

Supplemental material for "Attention and counter-framing in the Black Lives Matter movement on Twitter"

Colin Klein, Ritsaart Reimann, Ignacio Ojea Quintana,
Marc Cheong, Marinus Ferreira, and Mark Alfano

1 Data collection and preprocessing

1.1 Statement of Code and Data Availability

Python notebooks containing the entire processing chain for the paper along with instructions for adapting other datasets are available at `https://osf.io/amv3r/`. Also available are a collection of 'dehydrated' tweet IDs for both retweets and tweets, suitable for reconstruction using tools such as `twarc`.

1.2 Data Collection

Based on an initial snowball sampling of words, phrases, and hashtags associated with the Black Lives Matter movement conducted in 2013, we queried the Twitter Streaming API with a series of Black Lives Matter (BLM)-related keywords, hashtags, and short expressions in a window between January 1 2020 and December 31 2021.

The phrases used were: 'black lives matter', 'back the blue', 'back the badge', 'all lives matter', 'white lives matter', 'brown lives matter', 'take a knee', 'stand for our anthem', 'police brutality', 'blue lives matter'.

The hashtags used were: #BLM, #blacklivesmatter, #MassIncarceration, #BackTheBlue, #backthebadge, #AllLivesMatter, #WhiteLivesMatter, #BrownLivesMatter, #BlueLivesMatter, #HandsUpDontShoot, #SayHerName, #sayhisname, #ICantBreathe, #ICantBreath, #Policebrutality, #knowjusticeknowpeace, #thinblueline, #whosestreets, #takeaknee, #standforouranthem

1.3 Data Preprocessing

Tweets were preprocessed to remove non-alphabetic characters, URLs, and user mentions. Contractions, the *scikit-learn* English stopwords, web-specific words, and common articles in French and Spanish were removed. The remaining text was converted to lowercase and lemmatized (using *nltk*), retaining only words greater than 2 characters.

To build the LDA model, documents were used to build a `CountVectorizer` object and the model built on this representation.

1.4 The ACLED Dataset

The Armed Conflict Location & Event Data Project (ACLED) was used as our main data source of BLM-related events. ACLED (Raleigh et al., 2010) is a "disaggregated data collection, analysis, and crisis mapping project... [collating] the dates, actors, locations, fatalities, and types of all reported political violence and protest events around the world... [and] collects real-time data on political violence and demonstration activity across the entire world." (Armed Conflict Location & Event Data Project, 2022).

ACLED provides an 'export' tool to generate chronological datasets matching specified criteria. We restricted the analysis to events occurring in the United States in 2020 and 2021, and in which the `assoc_actor_1` field contained the string `BLM` ().

The complete list of data/metadata for each conflict event is documented at the ACLED Codebook. See `https://acleddata.com/acleddatanew//wp-content/uploads/2021/11/ACLED_Codebook_v1_January-2021.pdf`.

1.5 Missing Data

The Twitter API terms of service allow only one collection stream per API key. A misconfiguration error by the team meant that more than one colleciton used the same API key. As a result, our access to the Twitter API was cut off from July 24th through August 8th 2020. The misconfiguration did not affect the quality of the data collection itself, and after access was restored with the appropriate configuration there were no further disruptions. We have primary tweets from this time period if they were later retweeted, and we have retweets if they occur outside of this block.

For reasons explained in the main text, we think it unlikely that this gap will affect our primary results. Even using the academic API it is difficult to re-sample the missing gap with the same fidelity and expected volume as the original streaming API. However, we can estimate the effect it would have using another corpus covering the same time period.

To this end, we used the dataset offered by Giorgi et al. (2022), who collected a similar stream with a subset of the keywords we employed. While this should not be expected to get exactly the same set of authors, one would expect an overlap, and this provides a useful test of the robustness of our clustering to missing data. We rehydrated the list of ids for July and August 2020 using `twarc2` and rebuilt the retweet network using retweets that occurred during our

missing block. We then clustered the rebuilt network using the same algorithm and cutoff described in the main text.

		Rebuilt			
		Republican	Democrat	Activist	Not Present
	Republican	150817	3	0	478
Main	Democrat	5	134212	104	2125
Text	Activist	3	54	40515	330
	Not Present	662	5721	486	-

Figure 1: Group membership changes when adding additional data to cover gap.

Figure 1 compares membership between the two networks. As expected, groups were largely stable. There were very few authors who moved between groups; the primary changes involved nodes being added or deleted altogether. The largest change is with the Democrats, in which $\sim$ 1.5% of identified members dropped out of the network while about $\sim$ 4.2% of new nodes were added. None of the dropped members from Democrats were in the top 100 accounts by pagerank, suggesting that the differences are limited to less active accounts.

Topic modeling on a dataset that used different collection terms raises its own challenges, and we think it best to avoid refitting the models on alternative or resampled data. We note however that the analysis of the models is done in terms of proportions rather than raw counts, and that we have at least some tweet data from the missing gap so long as it was retweeted after the gap. The fact that there is no obvious discontinuity in the proportions in this stretch (even though there is far less data for the gap) suggests that the use of later retweets has not substantially distorted the analysis.

Finally, as we note in the main text, the autoregression code is natively adapted to account for missing data, and this was used in our procedure. One would expect missing data in this case to result in a small loss of statistical power, but this small loss does not seem sufficient to explain any observed null connections.

2 Additional Descriptive Statistics

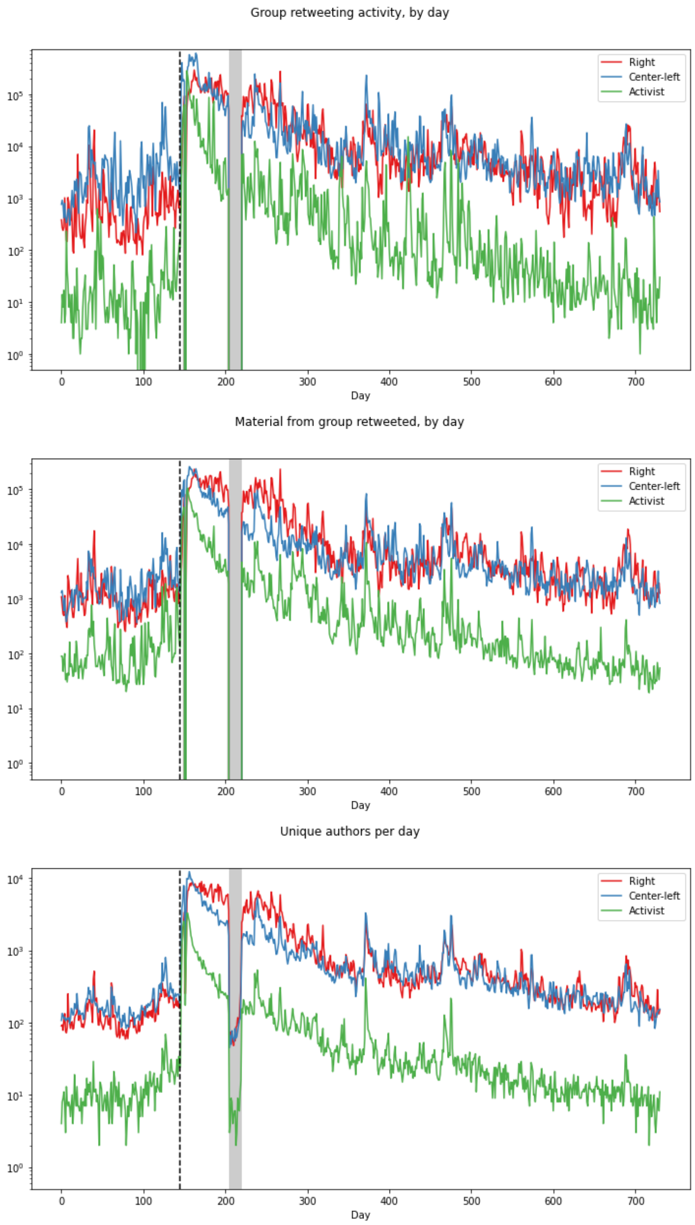

Figure 2: Retweets and unique author statistics by day. Dashed vertical line shows murder of George Floyd, gray bar shows gap in data collection.

Figure 3 compares the rate of data collection in our dataset versus that in the corpus provided by Giorgi et al. (2022). Giorgi et al. used fewer keywords than we used, only split data by months beginning in 2020, and provide only 9 months of data in 2020. Nevertheless, the shape of their monthly data rates in the overlapping months are broadly consistent with ours. Note that, post-Floyd, in both datasets, the number of *monthly* tweets exceeds that of all *years* of tweets before 2020.

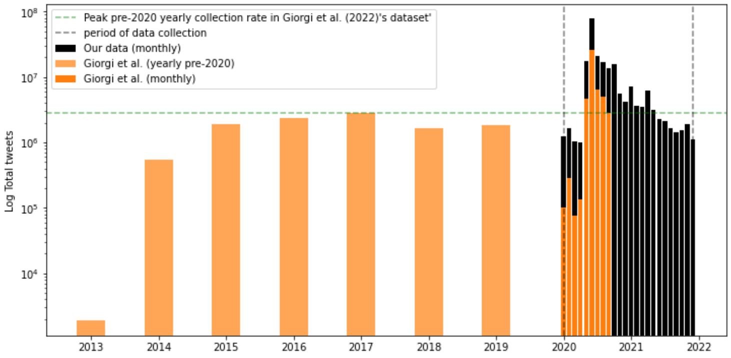

Figure 3: A comparison between our data collection rate and that of Giordi et al (2022).

3 Additional autoregression information

The z-transformed timeseries each had a significant AD Fuller test at $\alpha = 0.05$. Using the *statsmodels* function `adfuller` with a 1-day lag, p values were: Protests = 4.05×10^{-9}, Right = 0.011, Center-Left = 9.56×10^{-5}, Activist = 5.62×10^{-15}.

The full table of coefficients and p-values for the VAR are as shown in table 4:

Group/Variable	coef	std error	t-stat	p
Protests				
const	-0.002137	0.014118	-0.151	0.880
L1.protests	0.661429	0.024146	27.393	0.000
L1.Right	-0.080752	0.031774	-2.541	0.011
L1.Center-left	0.072957	0.051490	1.417	0.157
L1.Activists	0.295576	0.031607	9.352	0.000
Right				
const	0.000555	0.010965	0.051	0.960
L1.protests	0.026902	0.018753	1.435	0.151
L1.Right	0.797462	0.024678	32.315	0.000
L1.Center-left	0.213737	0.039991	5.345	0.000
L1.Activists	-0.068650	0.024548	-2.797	0.005
Center-left				
const	0.001603	0.011363	0.141	0.888
L1.protests	0.103263	0.019433	5.314	0.000
L1.Right	0.068430	0.025572	2.676	0.007
L1.Center-left	0.743144	0.041440	17.933	0.000
L1.Activists	0.084802	0.025438	3.334	0.001
Activist				
const	0.001869	0.017531	0.107	0.915
L1.protests	0.138879	0.029982	4.632	0.000
L1.Right	0.014360	0.039454	0.364	0.716
L1.Center-left	-0.077320	0.063936	-1.209	0.227
L1.Activists	0.832377	0.039247	21.209	0.000

Figure 4: Full table of VAR coefficients.

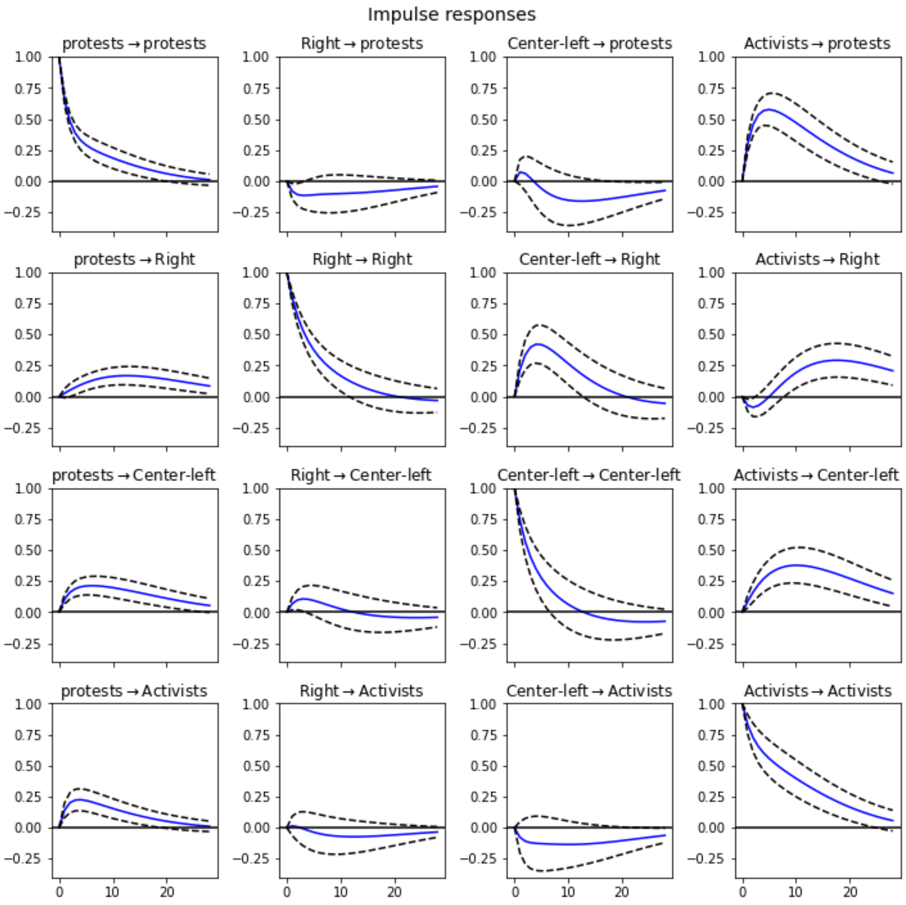

Figure 5: 28-day Impulse Response function for the full model. Dotted lines show 95% CI.

4 Topic Modeling

4.1 Main Results

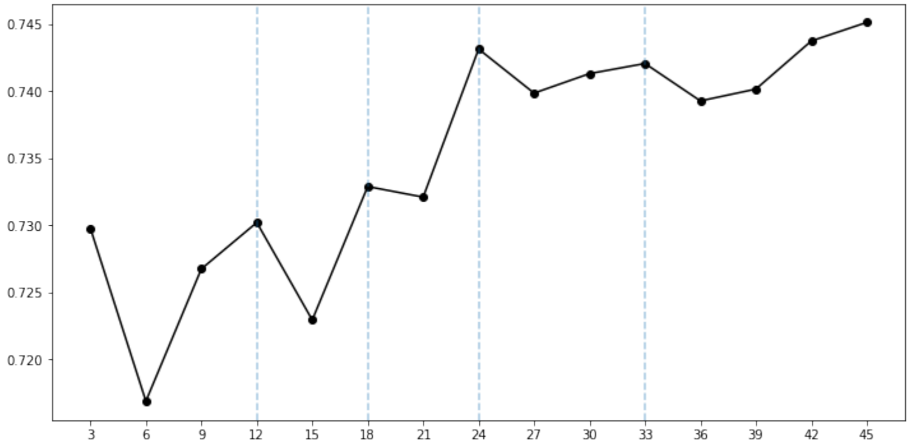

Figure 6: Group discriminability by number of topics. Baseline accuracy= 0.49. Vertical lines show local peaks.

#	Top words
0	blacklivesmatter georgefloyd breonnataylor black icantbreathe
1	matter black life live say white people movement racist support
2	blm antifa terrorist marxist riot black organization support
3	knee flag stand anthem nfl player national kneel watch sport
4	cop kill police murder officer shoot arrest man year black old
5	covid mask protest wear stay coronavirus news pandemic home
6	democrat biden party money fund joe soros democratic obama donation
7	vote trump american america president republican voter country
8	trump protest protester capitol say white sign police gun peaceful
9	medium social month america tell justice family remember really
10	police brutality protest black racism stop protester america
11	blm donate share retweet need facebook help joebiden face hate
12	history right human blacktwitter racism time change fix rap
13	blue help state red bring send business georgia donate flip
14	policebrutality video follow new watch anonymous check break
15	justice sayhername black today sign day george floyd fight breonna
16	people just say think white know make right fuck want racist
17	black racism make people issue movement end community white
18	trump maga usa kag wwgwga qanon patriot walkaway supporter lawandorder
19	police protest black protester trump street city activist mob
20	bluelivesmatter thinblueline say push slap jail report catholic
21	veteran update latino asian south mexican indian crash new american
22	alllivesmatter whitelivesmatter race color racist die skin alllivesmattter
23	backtheblue police god sayhisname officer law thank enforcement

Figure 7: Top words for 24-topic LDA model

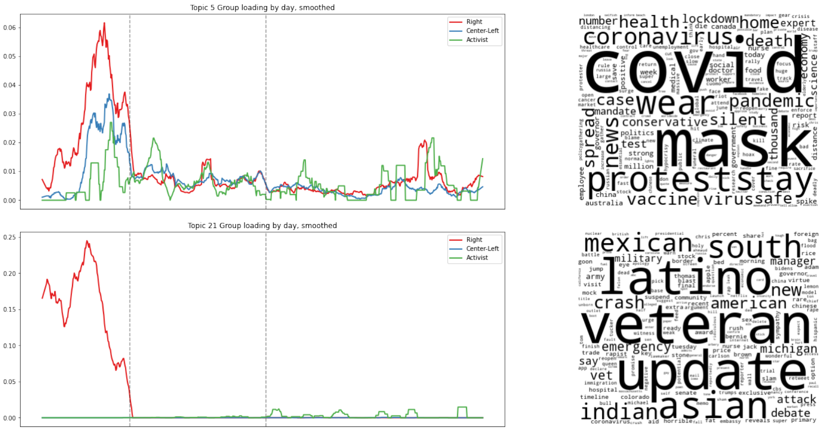

Figure 8: Proportion of tweets loading on topic 5 and 21

4.2 Topic modeling less Floyd-specific terms

A reviewer suggested that the topic modelling may have been driven partly by George Floyd-related terms. That is surely to be expected, and one of the dynamics that we sought to capture. Nevertheless, one might wonder what difference it would make to the models were those terms to be removed.

Note first that topic modelling is a stochastic process, and that different runs on the same dataset will tend to produce models which are similar but not identical. A useful—though strict—test of similarity between two runs is *symmetric cosine similarity* (SCS). To calculate this measure, first consider the nxn matrix representing the cosine similarity between each pair of topic-word vectors in n-topic models A and B. For each topic in A chose the topic B with the highest cosine similarity, and vice-versa. If the two choices match—that is if the topic most similar to n in A is m of B *and* the topic most similar to m of B is n in A—consider the topic to be reproduced. Calculate the overall similarity score for A and B as the number of matching topics. SCS is a strict measure because very often topics will resemble one another, or new topics will represent slight recombinations of older topics, which breaks symmetry even if the set of topics covers roughly the same themes.

We first evaluated the mean SCS between each of 20 new topic models run on the *same* corpus as the original: this gave a mean SCS of 19.15 ($SD = 3.12$). We then constructed 20 new 24-topic models on a corpus in which the terms 'floyd','george','georgefloyd', 'justiceforgeorgefloyd' and 'icantbreathe' were zeroed out in the count matrix on which the LDA was fit. (Zeroing ensures comparability of vocabulary and hence component matrices.) The result gave a mean SCS of 19.25 ($SD = 1.13$). In other words, the topic models which removed Floyd-related words were about as similar to the original model as re-runs of the original model were to each other.

Manual inspection of the topics suggested that the George Floyd related topic 0 was one of the less stable topics in the original. It was, unsurprisingly, one of the less likely to be replicated when Floyd-related words were removed. On the other hand, the other compared topics were generally as stable in the zeroed version as they were in the original, suggesting relative stability in revealed themes.

References

Armed Conflict Location & Event Data Project (2022). ACLED History. *Available at https://acleddata.com/acleddatanew//wp-content/uploads/dlm_uploads/2021/11/ACLED-History_v2_February_2022.pdf.*

Giorgi, S., Guntuku, S. C., Himelein-Wachowiak, M., Kwarteng, A., Hwang, S., Rahman, M., and Curtis, B. (2022). Twitter corpus of the #blacklivesmatter movement and counter protests: 2013 to 2021. *Proceedings of the International AAAI Conference on Web and Social Media*, 16(1):1228–1235.

Raleigh, C., Linke, A., Hegre, H., and Karlsen, J. (2010). Introducing ACLED: an armed conflict location and event dataset: special data feature. *Journal of peace research*, 47(5):651–660.
